# Predictive value of the Status Epilepticus Severity Score (STESS) and its components for long-term survival

**DOI:** 10.1186/s12883-016-0730-0

**Published:** 2016-11-05

**Authors:** Preben Aukland, Martin Lando, Ole Vilholm, Elsebeth Bruun Christiansen, Christoph Patrick Beier

**Affiliations:** 1 Department of Neurology, University Hospital of Odense, Sdr. Boulevard 29, 5000 Odense C, Denmark; 2 Department of Neurology, District Hospital Vejle, Vejle, Denmark; 3 Department for Clinical Research, University of Southern Denmark, Odense, Denmark; 4 Department of Neurology, University Hospital of Aachen, RWTH Aachen, Aachen, Denmark

**Keywords:** Epilepsy, Mortality, Long-term, Short-term, STESS

## Abstract

**Background:**

The “Status Epilepticus Severity Score” (STESS) is the most important clinical score to predict in-hospital mortality of patients with status epilepticus (SE), but its prognostic relevance for long-term survival is unknown. This study therefore examined if STESS and its components retain their prognostic relevance beyond acute treatment.

**Methods:**

One hundred twenty-five non-anoxic patients with SE were retrospectively identified in two hospitals between 2008 and 2014 (39.2 % refractory SE). Patients’ treatment, demographic data, date of death, aetiology of SE, and the components of the STESS (age, history of seizures, level of consciousness and worst seizure type) were determined based on the patients’ records.

**Results:**

In 94.4 % of patients, SE was treated successfully; in-hospital mortality rate was 12 %. The overall mortality was 42 % after median follow-up of 28.1 months. The survival plateaued after about 3 years, all patients with progressive brain diseases (*n* = 4) died within one year. In-hospital mortality correlated highly significantly with STESS, the optimal cut-off was 4. With respect to long-term outcome, STESS correlated significantly with overall mortality though with lower odds ratios. When looking only at patients that survived the acute phase of treatment, only the STESS components “level of consciousness” (at admission), “coma” as worst seizure type, and “age” reached a statistical significant association with mortality. In these patients, STESS with a cut-off of 4 was not significantly associated with survival/mortality. Aetiology of SE was insufficient to explain the weak association and the high mortality after discharge alone.

**Conclusion:**

STESS at onset of SE reliably assessed in-hospital mortality, and was indicative for overall survival. However, STESS did not allow correct estimation of mortality after discharge. The high mortality after discharge and high overall mortality of patients diagnosed with SE was not explained by progressive brain disorders alone. Further research is needed to understand the causes for high overall mortality after SE and putative prognostic factors.

**Electronic supplementary material:**

The online version of this article (doi:10.1186/s12883-016-0730-0) contains supplementary material, which is available to authorized users.

## Background

Status epilepticus (SE) is a serious neurological condition with significant acute mortality of 7–39 % and early treatment is of crucial importance [1–6]. The management and treatment of patients presenting with SE is widely debated. The treatment ranges from benzodiazepines, different anti-epileptic drugs to coma induction [7, 8]. Because of the clinical heterogeneity of the affected patients [9] and the lack of established prognostic factors, the prediction of the clinical outcome and survival of SE remains difficult. Rossetti et al. therefore developed the “Status Epilepticus Severity Score” (STESS, Additional file 1: Table S1) in the purpose to predict in-hospital mortality [10]. The score was designed to give the clinician an estimate of in-hospital mortality of each individual patient, based on four outcome predictors (“age”, “history of seizures”, “seizure type”, “extent of consciousness impairment”). With a maximum score of 6, Rossetti et al. found an optimal cut-off value at ≥3 with a sensitivity of 0.94 and specificity 0.60. Negative predictive value (NPV) was 0.97 and positive predictive value (PPV) was 0.39 [11]. STESS is a clinically used score to predict outcome after SE and has been externally validated in a second study [12]. In this confirmatory study, components “history of seizures” and “extent of consciousness impairment” but not “age” and “generalised convulsive seizures at SE onset” were significantly associated with higher odds for death. With a score of ≥4, the optimal cut-off for predicting in-hospital mortality was higher in this cohort [12].

Leitinger et al. recently developed a new “Epidemiology-based Mortality Status Epilepticus Score” (EMSE) that initially included a combination of six clinical parameters: aetiology, age, comorbidity, EEG, duration and level of consciousness. The authors concluded that the combination of aetiology, age, level of consciousness, +/−EEG (EMSE-EACE/EMSE-EAC) was in many ways superior to predict in-hospital mortality than STESS (≥3 and ≥4) [13]. However, a very recent study showed no significant difference between STESS and EMSE-EAC or EMSE-EACE [14].

With SE with respect to mortality and functional status, the long-term outcome after discharge of patients is essentially unknown. Hauser and co-workers studied a cohort of paediatric and adult patients surviving SE at least for 30 days. They followed them until death or end-of-study and found a long-term mortality of 40 % [15]. Ristic et al. reported a mortality rate of 22.2 % in a cohort of patients treated in a tertiary reference centre. Unfortunately, follow up data was available for only 32.8 % of the surviving patients [16]. Apart from patients with progressive neurological diseases (typically brain tumours), it is often unknown why patients die several months after SE. If and how the consequences of prolonged SE, e.g. neuronal death due to excitotoxicity or alteration of neuronal networks, contribute to the high mortality is unknown [9]. Given that SE treatment often includes treatment at intensive care units (ICU), which is associated with significant mortality [17, 18], prognostic factors and scores allowing determining long-term survival after SE are of high importance.

This study therefore aimed at determining the accuracy of STESS on long-term survival based on a population of patients presenting with SE at admission or during hospital stay, treated in two academic centres in Southern Denmark.

## Methods

### Patients and ethics

All identifiable patients with SE who have been treated at the Regional Hospital of Vejle (August 2008 – October 2013) and the University Hospital of Odense (August 2008 to March 2014) were included. Both hospitals are regional referral centre for patients with SE. The study was approved by the local and national authorities for data security (Sundhedsstyrelsen, 3-3013-696/1) and evaluated by the local ethics committee. The adult patients (≥18 years) were retrospectively identified based on ICD-10 codes at discharge (G41X) or documented SE in the patient records.

#### Inclusion criteria

On-going clinical or EEG-verified seizures for more than five minutes or repetitive seizures without normalization of consciousness in-between [7]

Age of 18 or older

#### Exclusion criteria

Patients younger than 18

An-/hypoxic-ischemic encephalopathy (*n* = 10)

The patients’ records were retrospectively analysed. The patients’ journals were used to score each patient according to STESS [10]. In addition, aetiology (categorized as proposed by the International League Against Epilepsy, ILAE [19]) was assessed. In-hospital mortality was defined as death under acute treatment. Patients discharged from hospital to ambulant palliative care units were considered as “survivors of acute treatment”. Refractory patients were defined as patients that did not show prompt response to first- and second-line treatment with phenytoin, valproate, phenobarbital, or levetiracetam. The mortality after discharge was determined using the date of death registered in the Danish Civil Register, available in the patient records of deceased patients.

### Statistics

The primary, predefined outcome of the study was STESS’ ability to predict in-hospital and overall mortality of SE patients. To determine differences of the prognostic impact of the STESS components Odds ratio and Pearson’s Chi-square were used. The 95 % confidence interval (CI) of Odd’s ratio was determined as described in [20]. Table 1 was analyzed using Chi-square tests, Fisher’s exact test, and student-*t* test. P-values ≤0.05 were considered significant, values were not corrected for multiple testing. The ability of STESS to separate survivors from non-survivors in the acute phase and after discharge was illustrated using receiver-operating characteristic curves (ROC). The optimal cut-off point of the STESS regarding sensitivity and specificity for prediction of death were calculated using Youden’s index (Youdens index J = Sensitivity + Specificity – 1). The confidence interval (CI) of ROC was calculated as described in [21]. Sensitivity and specificity was found and used in calculating NPV and PPV at each cut-off. Long-term survival was addressed using Kaplan-Meier estimator and compared using log-rank test. Statistical analyses were performed using SPSS 22.0 and Microsoft Excel.

**Table 1:** Patient population

Characteristics	Total cohort	Non-survivors	Survivors	P-value
	*n* = 125 (%)	*n* = 58 (42 %)	*n* = 67 (58 %)	
Gender, n (%)				
Male	64 (51)	36 (62)	28 (42)	0.02*
Female	61 (49)	22 (38)	29 (58)	
Age, year (mean ± SD)	62.9 ± 17.5	69.8 ± 15.7	56.9 ± 16.8	<0.01§
SE aetiology grouped according to the international League Against Epilepsy, n (%)				
Acute symptomatic seizures	48 (38)	27 (46)	21 (31)	0.16*
Remote symptomatic unprovoked seizures	26 (21)	9 (16)	16 (24)	
Symptomatic seizures due to progressive CNS disorders	4 (3)	3 (5)	1 (2)	
Unprovoked seizures of unknown aetiology	47 (38)	19 (33)	29 (43)	
Refractory SE	49 (39)	29 (50)	20 (30)	0.02*
Seizure termination, n (%)	118 (94)	51 (88)	67 (100)	0.003#
Narcosis	27 (22)	15 (26)	12 (18)	0.28*
STESS at onset of SE, n (%)				
Level of consciousness				
Awake/somnolent	67 (54)	22 (38)	45 (67)	0.01*
Stuporous/comatose	58 (46)	36 (62)	22 (33)	
Worst seizure type				
Simple or complex/absence	69 (55)	14 (24)	23 (34)	0.02*
Generalized convulsive	37 (30)	28 (48)	41 (61)	
NCSE in coma	19 (15)	16 (28)	3 (4)	
Age ≥ 65 years	60 (48)	37 (63)	23 (34)	0.001*
History of seizures	75 (60)	27 (46)	23 (34)	0.16*
STESS ≥ 3	64 (51)	46 (79)	18 (27)	<0.001
STESS ≥ 4	82 (66)	55 (95)	27 (40)	<0.001

*Chi-square test

#Fisher’s exact test

§*t*-test

## Results

### Patient demographics

The demographics and baseline characteristics of all included patients are summarized in Table 1. The mean time of follow up was 30 months (range: 1.5–75.5 months); median follow-up was 28.1 months. The aetiology was classified according to the ILAE categorization [19] and spread among the categories, though very few of the patients had symptomatic seizures due to progressive CNS disorders. Data on STESS components (Additional file 1: Table S1), outcome at end-of-study, outcome at discharge, and acute treatment (incl. narcosis) were available for all patients.

### STESS and in-hospital mortality

Seizures could be terminated in 94.6 % of the patients. 22 % of the patients were treated in the ICU with deep sedation typically resulting in a burst-suppression pattern in the EEG (Table 1). By the time of discharge 12 % of the patients died (Table 2A). Of these 15 patients, 7 patients died due to refractory SE, where the seizures could not be terminated. 8 patients died after SE because of complications or from underlying SE aetiology. The complications that led to death were sepsis (2 patients) and multiple organ failure (1 patient). The underlying fatal SE aetiologies were encephalitis (1 patient), intracranial haemorrhage (2 patients), meningitis (1 patient), and brain tumour (1 patient).

**Table 2:** STESS components of survivors and non-survivors

Analysis of survival at discharge/in-hospital mortality				
A. STESS at Onset of SE	Survivors (*n* = 110)	Non-survivors (*n* = 15)	Odds Ratio (95 % CI)	*P*-value
Level of consciousness n(%)				
Awake/somnolent	64(58)	3(20)	Reference	
Stuporous/ comatose	46(42)	12(80)	5.6 (1.4–21)	0.006
Worst seizure type n(%)				
Simple or complex/absence	35(32)	2(13)	Reference	
Generalized convulsive	66(60)	3(20)	0.8 (0.1–4.9)	0.80
NCSE in coma	9(8)	10(67)	19.4 (3.6–104)	0.0001
Age n(%)				
< 65 years	61(55)	4(27)	Reference	
≥ 65 years	49(45)	11(73)	3.4 (1–11.4)	0.03
History of seizures n(%)				
Prior seizures	71(65)	4(27)	Reference	
No prior seizures	39(35)	11(73)	5.0 (1.5–16.7)	0.005
Analysis of overall survival at end-of-study				
B. STESS at Onset of SE	Survivors (*n* = 67)	Non-survivors (*n* = 58)	Odds Ratio (95% CI)	P-value
Level of consciousness. n(%)				
Awake/somnolent	45(67)	22(38)	Reference	
Stuporous/ comatose	22(33)	36(62)	3.4 (1.6–7)	0.001
Worst seizure type. n(%)				
Simple or complex/absence	23(35)	14(24)	Reference	
Generalized convulsive	41(61)	28(48)	1.1 (0.5–2.5)	0.78
NCSE in coma	3(4)	16(28)	8.7 (2.2–35.6)	0.001
Age. n(%)				
< 65 years	44(66)	21(36)	Reference	
≥ 65 years	23(34)	37(64)	3.4 (1.6–7)	0.001
History of seizures. n(%)				
Prior seizures	44(66)	31(53)	Reference	
No prior seizures	23(34)	27(47)	1.7 (0.8–3.4)	0.16
Analysis of survival of patients that survived acute SE				
C. STESS at Onset of SE	Survivors (*n* = 67)	Non-survivors (*n* = 43)	Odds Ratio (95 % CI)	P-value
Level of consciousness. n(%)				
Awake/somnolent	45 (67)	19(44)	Reference	
Stuporous/ comatose	22 (33)	2456)	2.6 (1.2–5.7)	0.017
Worst seizure type. n(%)				
Simple or complex/absence	23(34)	12(28)	Reference	
Generalized convulsive	41(63)	25(58)	1.2 (0.5–2.8)	0.72
NCSE in coma	3(3)	6(14)	3.8 (0.8–18)	0.08
Age. n(%)				
< 65 years	44(66)	17(40	Reference	
≥ 65 years	23(34)	26(60)	2.9 (1.3–6.4)	0.007
History of seizures. n(%)				
Prior seizures	44(66)	27(63)	Reference	
No prior seizures	23(34)	16(37)	1.1 (0.5–2.5)	0.76

**Fig. 1:**
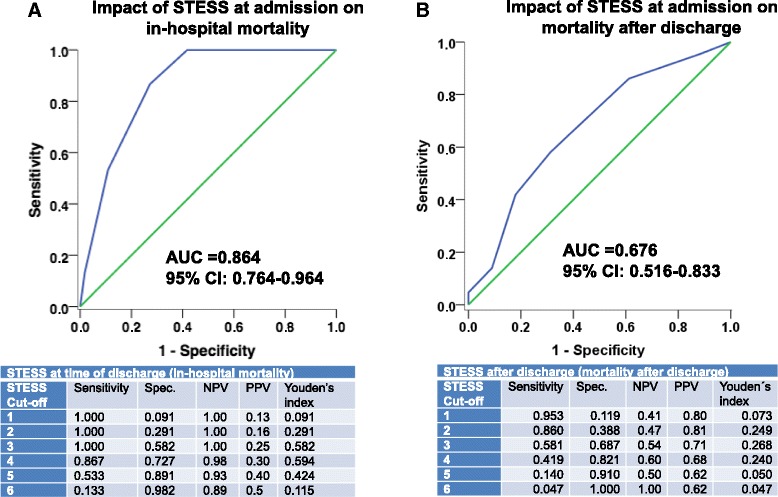
**a**-**b** ROC plotting sensitivity versus 1-specificity for each cut-off value with a diagonal reference line. The table below each ROC curve gives sensitivity, specificity, NPV, PPV, and Youdens index. **a** ROC for patients’ death under treatment in the hospital (in-hospital mortality) **b** ROC for patients that were discharged alive from hospital

All components of STESS were significantly associated with higher odds for death at the time of discharge (Table 2A). Notably, in the category “worst seizure type”, no differences between patients presenting with generalized convulsive seizures and simple/complex-partial seizures were found. Patients presenting in coma (i.e. without apparent clinical seizures) had a substantially increased mortality. Determination of Youden’s index identified 4 as optimal cut-off in this patient cohort (Fig. 1a). Of the patients with a STESS less than 4, 80/82 patients survived, while 12/42 patients with STESS ≥ 4 died. This gave a high negative predictive value of 98 % and a positive predictive value of 30 % (Fig. 1a).

### STESS and survival after discharge

**Fig. 2:**
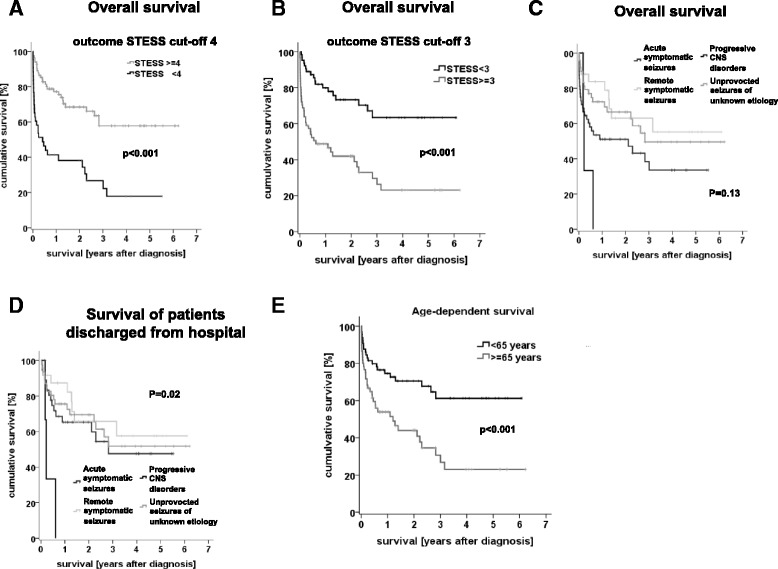
Kaplan-Meier estimator of survival. The small vertical tick-marks indicate patients that were alive at the time point of analysis. **a** Kaplan-Meier plot presenting overall survival at end-of-study for patients with a favourable STESS 0–3 (*grey line*) and an unfavourable STESS of 4–6 (*black line*). **b** Kaplan-Meier plot presenting overall survival at end-of-study for patients with a favourable STESS 0–2 (*grey line*) and an unfavourable STESS of 3–6 (*black line*). **c** Kaplan-Meier plot presenting overall survival depending on aetiology. **d** Kaplan-Meier plot presenting survival depending on aetiology of all patients that were discharged alive from hospital. **e** Kaplan-Meier plot presenting overall survival depending on age at diagnosis above or below 65 years

After a median follow-up of 28.1 months, the overall mortality was 42 % (in-hospital mortality: 12 %). The prognostic significance of STESS for long-term outcome has not been studied yet. We therefore analysed the association of STESS and its components with overall survival. The components of STESS were significantly associated with higher odds for death at end-of-study, except for history of previous seizures (Table 2B). Still, the Odds’ ratios for overall mortality at end-of-study were lower than for at the time of discharge (“in-hospital mortality”). “Worst seizure type” remained highly predictive for survival but the differentiation between “generalized convulsive seizures” and “simple/complex-partial seizures” remained without prognostic significance. Determination of Youdens’ index showed that a cut-off for analysis of 3 was slightly better than a cut-off of 4. Using 3 as cut-off, the calculated NPV of 72 % was considerably lower than at discharge, the PPV was 66 %. The sensitivity of STESS ≥ 3 for bad outcome was 0.69, the specificity 0.31 (Additional file 1: Table S2). When using the optimal cut-off for in-hospital (STESS ≥ 4) survival, the average survival time was 48.7 months (CI: 40.9–56.5 months) and significantly longer than for patients with a non-favourable score (19.6 months, CI: 11.5–29.4 months, *p* < 0.001, Fig. 2a; b shows the same analysis with a cut-off of ≥3). The difference between patients with a favourable and non-favourable STESS became even more pronounced when looking at patients with refractory SE only (Additional file 2: Figure S1).

The analysis of overall survival includes both, in-hospital mortality and death after discharge from hospital. The mortality of the 110 patients that survived acute treatment in the hospital was 39.1 %. To study if the single components of STESS retained prognostic relevance beyond the acute phase of treatment, we analysed the outcome of patients that survived acute treatment of SE and could be discharged alive from hospital. With a cut-off of 4, STESS was not associated any more with survival after discharge (*p* = 0.29). The STESS components (assessed at onset of SE) “age”, “level of consciousness”, and worst seizure type “coma at onset of SE” remained associated with long-term outcome though with a low odds ratio (Table 2C). “Previous seizures” and the differentiation between “generalized convulsive seizures” and “simple/complex-partial seizures” as “worst seizure type” were without prognostic relevance (Table 2C). Figure 1b shows ROC curve for patients that could be discharged from hospital, which illustrates the low sensitivity and specificity for patients’ survival after discharge.

### Aetiology and long-term survival

Although components of STESS remained predictive for the overall survival of SE patients also after discharge, STESS (cut-off 4) did not reach statistical significance. In contrast to e.g. the EMSE, STESS does not directly consider SE aetiology, which may be one possible cause for the failure of STESS to predict survival after discharge of the hospital [13]. We therefore performed an additional, exploratory study on the impact of aetiology on outcome after SE. When looking at overall survival of the total population, we could see differences depending on the underlying aetiology (defined according to ILAE categorisation): All 4 patients suffering from progressive diseases died rapidly (Fig. 2c). The patients with seizures due to unknown aetiology showed intermediate survival, while patients belonging to the other groups (acute, symptomatic seizures, remote, symptomatic, unprovoked seizures) showed similar, better long-term outcome. Notably, the differences between patients with unknown aetiology and the two other groups with more favourable outcome disappeared when looking at patients that survived acute phase of SE (Fig. 2d), mainly due to the high mortality of patients with acute symptomatic seizures (11 out of 15 patients that died in the hospital). Still, the mortality of all patients remained high and plateaued after about 3 years (Fig. 2c, D). Further, we looked at the impact of age on the overall outcome given that a substantial mortality is not uncommon in high age. The survival curves of patients above and under the age of 65 years significantly differed but showed the same plateau after about 3 years (Fig. 2e).

## Discussion

An important finding of this study is the high mortality after discharge. After an average follow-up of 30 months, 42 % of our patients died. Our mortality and the data published by Logroscino et al. appear to be in the same range despite of the differences in the patient populations. Logroscino et al. analysed 145 patients (including paediatric patients) that survived 30 days after diagnosis of SE and found a 10-year mortality of 43 % [15]. These rates were substantially higher than the mortality rates of 22.2 % described by Ristic et al. [16]. However, Ristic et al. reported incomplete follow-up data in more than 2/3 of the patients, which may explain this difference.

The high mortality also prompts the question if our cohort was biased. We assume that the study population included all patients that required intravenous 2nd line treatment as well as all patients with refractory (refractory to 2nd line treatment) and super-refractory SE (refractory to narcosis), because all these patients are treated in the neurological departments. However, it is likely that an unknown number of patients with prompt success of 1st line treatment with benzodiazepines (e.g. patients with SE due to alcohol withdrawal) were not included in this study due to incorrect use of ICD-10 codes in the emergency departments. However, this potential minor bias did not lead to a higher proportion of severely ill patients included in this study and does therefore not explain the high overall mortality after SE in our cohort. Our cohort comprised 40 % patients with refractory SE (defined by failure of 2nd line therapy), which is exactly the same proportion as reported in comparable studies [10, 12, 13, 22]. Comparing the patient demographics and baseline characteristics to the first study made by the STESS inventors [10] was neither indicative for a substantial selection bias. Our cohort had a lower proportion of acute symptomatic aetiology (38 % vs. 56 %) but included slightly more elderly patients (48 % vs. 35 % ≥65 years). The in-hospital mortality of our patients was actually lower (12 %) than in similar studies by Sutter et al. (18 %) or Rosetti et al. (21 %) [10, 12], showing that selection bias does not explain the high mortality.

The analysis of the underlying aetiology and the analysis of patients with refractory SE only (Additional file 2: Figure S1) did neither provide an obvious explanation for the poor outcome of the patients. The same applies for an obvious flaw of our study, the very variable follow-up: it may even have masked a higher morbidity. In summary, we think that the high overall mortality of patients with SE is a matter of fact and not due to selection bias.

In the light of the very high mortality of patients with SE after discharge, the prediction of outcome beyond acute treatment becomes as important as in-hospital mortality. No previous studies have tested, if STESS (or other clinically relevant scores) predicts long-term survival. Our study confirmed the prognostic significance of the STESS for in-hospital mortality in this Danish cohort of patients [10, 12]. STESS with a threshold of 4 reliably identified patients that survived the acute phase of SE, and predicted to some extend in-hospital mortality. We here complement current knowledge by showing for the first time that STESS also predicts overall mortality (though with a lower sensitivity and specificity) but is not significantly associated with mortality after discharge. The Kaplan-Meier-plot based on the optimal cut-off of ≥ 4 in the discharge analysis displays this significant difference in overall mortality (Fig. 2a).

All components of the STESS except “history of previous seizures” remained significantly associated with long-term survival and survival after discharge. This indicates “history of previous seizures” is solely relevant for the acute phase of SE, possibly because patients with acute symptomatic seizures (and no history of previous seizures) have higher odds of dying in the hospital. Of note, the differentiation between seizure types remained without prognostic relevance in all our analyses in line with the report by Sutter et al. [12].

Among all components, “coma” yielded most prognostic information but only relevant for a few patients. Coma had higher odds for death (in-hospital mortality: 19.4 and overall mortality: 8.8) than STESS (in-hospital mortality: 17.3, overall mortality: 5.3) in all our analyses, suggesting that this factor may even warrant a higher score. Only 3 out of 19 patients presenting with “coma at onset” of SE were alive at end-of-follow up.

The other major factor with similar importance was “age above 65”, which gave 2 points in the STESS. The Kaplan-Meier curves of patients above and below 65 years (Fig. 2e) were similar to the Kaplan-Meier curves of patients with STESS ≥3 (or STESS ≥4, Fig. 2b). However, neither the high mortality of SE patients nor the plateau after approximately 3 years can be explained by age alone or by naturally occurring death of the patients. Assuming that natural occurring death in higher age would explain the difference alone, one would not expect a major difference 6 months after SE. However, this analysis (Additional file 1: Table S3) revealed similar results as the analysis at the end-of-study. Further, the Kaplan-Meier curves of patients with STESS ≥3 (or STESS ≥4) show a much more rapid decline than the curve of patients above and below 65, which further supports the idea that STESS bears more prognostic information than age alone.

It is tempting to speculate that neuronal damage after SE may be the factor that substantially contributes to overall outcome of patients with SE. In patients with acute neuronal damage (like stroke [23]), age is a major prognostic factor and neuronal damage may therefore explain the high relevance of age as prognostic factor. However, this retrospective study with all its limitations (e.g. mortality as only outcome parameter, lack of detailed assessment of functional outcome, retrospective design, incomplete assessment of other prognostic factors, etc.) does not provide clear clues in support of this hypothesis and further studies are required to better understand the important contributors to long-term survival after SE beyond aetiology.

## Conclusions

In summary, this study supports STESS as a valuable and easy-to-use tool to estimate in-hospital mortality. The overall mortality after SE was found higher than expected compared to previous research. Most of STESS’ components were found to have an association to the overall mortality after SE. There are though reasons to believe that other factors may play important roles for long-term outcome. A prognostic score for long-term survival may be based on STESS but will certainly require modifications to improve its ability to predict long-term survival.

## Additional files

Additional file 1:
**Tables S1–S3.** (DOC 63 kb)
Additional file 2: Figure S1. Kaplan-Meier plot presenting overall survival at end-of-study for patients with refractory (black line) and non-refractory (grey line) SE. The small vertical tick-marks indicate patients that were alive at the time point of analysis. (PDF 213 kb)

## Abbreviations

EEG: Electroencephalography
EMSE: Epidemiology-based Mortality Score Status Epilepticus
ILAE: International League Against Epilepsy
NPV: Negative predictive value
PPV: Positive predictive value
ROC: Receiver-operating characteristic curve
SE: Status epilepticus
STESS: Status Epilepticus Severity Score
