# Supplementary material for: Predictive value of the Status Epilepticus Severity Score (STESS) and its components for long-term survival
Source: BMC Neurol. 2016 Nov 5;16:213. doi: 10.1186/s12883-016-0730-0 (PMC5097843; doi:10.1186/s12883-016-0730-0)
Supplement: Additional file 1: — Tables S1–S3. (DOC 63 kb) [file 12883_2016_730_MOESM1_ESM.doc]

Additional file 1

**Table S1: STESS**

|  | **Features** | **STESS** |
| --- | --- | --- |
| **Consciousness** | Alert or somnolent/confused | 0 |
|  | Stuporous or comatose | 1 |
| **Worst seizure type** | Simple-partial, complex-partial, absence,  myoclonic | 0 |
|  | Generalized-convulsive | 1 |
|  | Nonconvulsive status epilepticus in coma | 2 |
| **Age** | < 65 years | 0 |
|  | ≥ 65 years | 2 |
| **History of previous**  **Seizures** | Yes | 0 |
|  | No or unknown | 1 |

**Table S2:** STESS and overall mortality

|  | | | | | |
| --- | --- | --- | --- | --- | --- |
| **STESS**  **Cutoff** | Sensitivity | Specificity | NPV | PPV | Youden´s  index |
| **1** | 0.966 | 0.119 | 0.80 | 0.49 | 0.085 |
| **2** | 0.897 | 0.388 | 0.81 | 0.57 | 0.285 |
| **3** | 0.690 | 0.313 | 0.72 | 0.66 | 0.377 |
| **4** | 0.534 | 0.821 | 0.67 | 0.72 | 0.355 |
| **5** | 0.241 | 0.91 | 0.58 | 0.70 | 0.151 |
| **6** | 0.069 | 1 | 0.53 | 1 | 0.061 |

**Table S3: Analysis of survival 6 months after SE**

|  | | | | |  |
| --- | --- | --- | --- | --- | --- |
| **STESS at Onset of SE** | Survivors  (n=81) | Non-survivors  (n=37) | Odds Ratio (95% CI) | P-value | |
| **Level of consciousness n(%)** |  |  |  |  | |
| **Awake/somnolent** | 50(62) | 12(32) | Reference |  | |
| **Stuporous/ comatose** | 31(38) | 25(68) | 3.4 (1.5-7.3) | 0.003 | |
| **Worst seizure type. n(%)** |  |  |  |  | |
| **Simple or complex/absence** | 26(32) | 7(19) | Reference |  | |
| **Generalized convulsive** | 50(62) | 16(43) | 1.2 (0.4-3.2) | 0.73 | |
| **NCSE in coma** | 5(6) | 14(38) | 10.4 (2.7-38.9) | 0.0002 | |
| **Age. n(%)** |  |  |  |  | |
| **< 65 yr** | 48(59) | 13(35) | Reference |  | |
| **≥ 65 yr** | 33(41) | 24(65) | 2.7 (1.2-6.0) | 0.01 | |
| **History of seizures. n(%)** |  |  |  |  | |
| **Prior seizures** | 51(63) | 18(49) | Reference |  | |
| **No prior seizures** | 30(37) | 19(51) | 1.8 (0.8-3.9) | 0.14 | |
