# Supplementary figures and images for: Predictive value of the Status Epilepticus Severity Score (STESS) and its components for long-term survival
Source: BMC Neurol. 2016 Nov 5;16:213. doi: 10.1186/s12883-016-0730-0 (PMC5097843; doi:10.1186/s12883-016-0730-0)

# Supplementary Figure 1

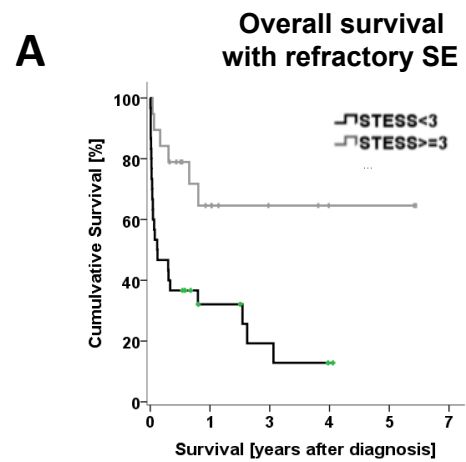

Supplement: Additional file 2: Figure S1. — Kaplan-Meier plot presenting overall survival at end-of-study for patients with refractory (black line) and non-refractory (grey line) SE. The small vertical tick-marks indicate patients that were alive at the time point of analysis. (PDF 213 kb) [file 12883_2016_730_MOESM2_ESM.pdf]
